# Development and Feasibility Study of Sustainable ePlate: A Web-Based Serious Game for Multidimensional Sustainable Healthy Diet Interventions in Schools

**DOI:** 10.3390/nu18172812

**Published:** 2026-08-27

**Authors:** Elena Patra, Maria Hassapidou, Odysseas Androutsos, Christos Diou, Ioannis Pagkalos

**Affiliations:** 1 Nutrition Information Systems Laboratory (NISLAB), Department of Nutritional Sciences and Dietetics, International Hellenic University, 57400 Thessaloniki, Greece; 2 Laboratory of Clinical Nutrition and Dietetics, Department of Nutrition and Dietetics, School of Physical Education, Sports Science and Dietetics, University of Thessaly, 42132 Trikala, Greece; 3 Department of Informatics and Telematics, School of Digital Technology, Harokopio University of Athens, 17778 Athens, Greece

**Keywords:** serious game, sustainable healthy diet, nutrition education, behavioral monitoring, gamification, school intervention, digital interventions

## Abstract

Background: School-based sustainable and healthy diet (SHD) interventions often inadequately capture the multidimensional nature of sustainability, with socioeconomic and cultural dimensions frequently underrepresented. While digital interventions can facilitate the integration of multiple SHD components, maintaining user engagement remains a persistent challenge that can compromise intervention effectiveness. Digital serious games represent a promising intervention strategy that can address both limitations by delivering multidimensional SHD content through interactive and engaging learning, while enabling real-time behavioral monitoring. Objectives: To assess the feasibility, as well as the acceptability and user experience of “Sustainable ePlate”, a web-based serious game for multidimensional SHD educational interventions in schools, during school-based sessions. Methods: Sustainable ePlate integrates food-level sustainability indicators into a scaffolded nine-challenge serious game. Challenges are presented through the healthy eating plate structure, while addressing eight food-level sustainability indicators transformed into 1–5 ordinal game scores. A database of 138 food items was developed from publicly available LCA and TCA databases. The game design draws on established learning theories implemented through progressive challenge sequencing, tiered feedback, and social features. Feasibility was assessed in guided school sessions in Northern Greece using the eGameFlow questionnaire and system in-app metrics. Results: The assessment sample comprised *n* = 59 students from two secondary schools, of whom *n* = 57 completed the eGameFlow questionnaire. Mean overall enjoyment was 82.54 ± 16.33%. Mean session duration was 22.39 ± 12.68 min. Across all sessions, 6003 food selections were recorded, with 5179 correct and 824 incorrect placements, corresponding to an 86.27% placement accuracy rate. In-app metrics indicated strong technical feasibility, with all sessions completing the full game sequence. Behavioral metrics demonstrated active interaction, characterized by placement interval duration, hesitation times, and high placement accuracy. Conclusions: Sustainable ePlate demonstrated feasibility and a positive user experience as a school-based serious game for multidimensional SHD education, and as a research platform for capturing in-app behavioral metrics. Further studies are required to assess its educational effectiveness in improving knowledge, attitudes, and practices related to sustainable healthy diets through pre–post design interventions.

## 1. Introduction

The global food system faces escalating challenges at the intersection of human and planetary health, including rising rates of diet-related non-communicable diseases, such as obesity or being classed as overweight [1], alongside significant environmental pressures from food production [2,3,4]. Sustainable healthy diets (SHDs) have emerged as a multidimensional concept that integrates nutritional adequacy, environmental impact, and socio-cultural factors [5,6,7,8,9,10]. Due to their multidimensional nature, SHDs are difficult to integrate in classic educational contexts, where simplified representations often fail to capture trade-offs between sustainability dimensions.

This reinforces the need for educational strategies translating complex sustainability concepts into accessible and actionable knowledge targeting younger populations during critical habit-formation years [11,12,13]. School-based interventions, while a key environment for SHD interventions [14,15,16,17], often simplify sustainability concepts and inadequately reflect the economic, social, and cultural dimensions of dietary sustainability [11,18,19,20]. Although some food system game approaches address multiple components [21], a 2023 systematic review on the focus of SHD interventions in schools [22] showed that sustainability coverage was narrow and uneven, more specifically in such interventions social, economic, and cultural dimensions are substantially underrepresented, indicating a structural mismatch between the multidimensional SHD concept and educational practice [8,23], leaving students with a fragmented understanding of food system sustainability.

Traditional school-based nutrition interventions also lack mechanisms to capture real-time engagement during activities, with evaluation relying on self-reported assessment. The significance of this engagement gap becomes clearer when considered alongside evidence from digital nutrition interventions which have demonstrated effectiveness in improving dietary knowledge, attitudes, and food choice behaviors [24], with effectiveness consistently moderated by design quality: theory-driven interventions incorporating behavior change techniques (BCTs) such as goal setting, real-time feedback, and self-monitoring produce larger and more sustained effects than those relying on information provision alone [25,26]. Serious games—digital applications designed with educational, health, or social objectives as their primary purpose [27]—can address both the content and engagement challenges above, supporting active learning while generating detailed user interaction data. Systematic reviews and meta-analyses confirm that game-based interventions can improve nutritional knowledge, attitudes, and short-term dietary behaviors in school-aged populations [25,28,29,30,31,32,33]. However, most existing systems do not incorporate multidimensional sustainability modeling or comprehensive behavioral monitoring [21], highlighting the need for digital tools that integrate the full breadth of SHDs, while enabling detailed monitoring of in-app user interaction data.

Nutrition-focused serious games have shown potential for improving short-term nutritional knowledge and selected dietary behaviors in children and adolescents, although findings remain heterogeneous and long-term retention is infrequently assessed [34]. Representative interventions such as FoodRateMaster [35] and Foodbot Factory [36] have combined game-based nutrition education with objective knowledge assessment, illustrating the value of separating user-experience outcomes from educational outcomes. Serious games addressing food system sustainability have more frequently focused on individual topics, such as food waste, environmental impact, or selected food system trade-offs [37]. A 2025 review identified 19 games covering at least two food system components, eight of which addressed sustainable food system outcomes, and found that only two had the potential to cover nearly all components [21]. Furthermore, the overall gap is not the complete absence of multidimensional food system games, but the scarcity of school-oriented tools that integrate nutritional, environmental, socioeconomic, and cultural considerations.

To address the above, we developed Sustainable ePlate, a web-based serious game designed for multidimensional SHD education in schools. The main objective was to evaluate feasibility—encompassing user acceptability, user experience, and successful capture of in-app interaction metrics—in a real school setting, as well as to describe the platform’s design, including its food database, sustainability-scoring framework, and gamification logic.

## 2. Materials and Methods

### 2.1. Development Approach

#### 2.1.1. Overview

**Figure 1:**
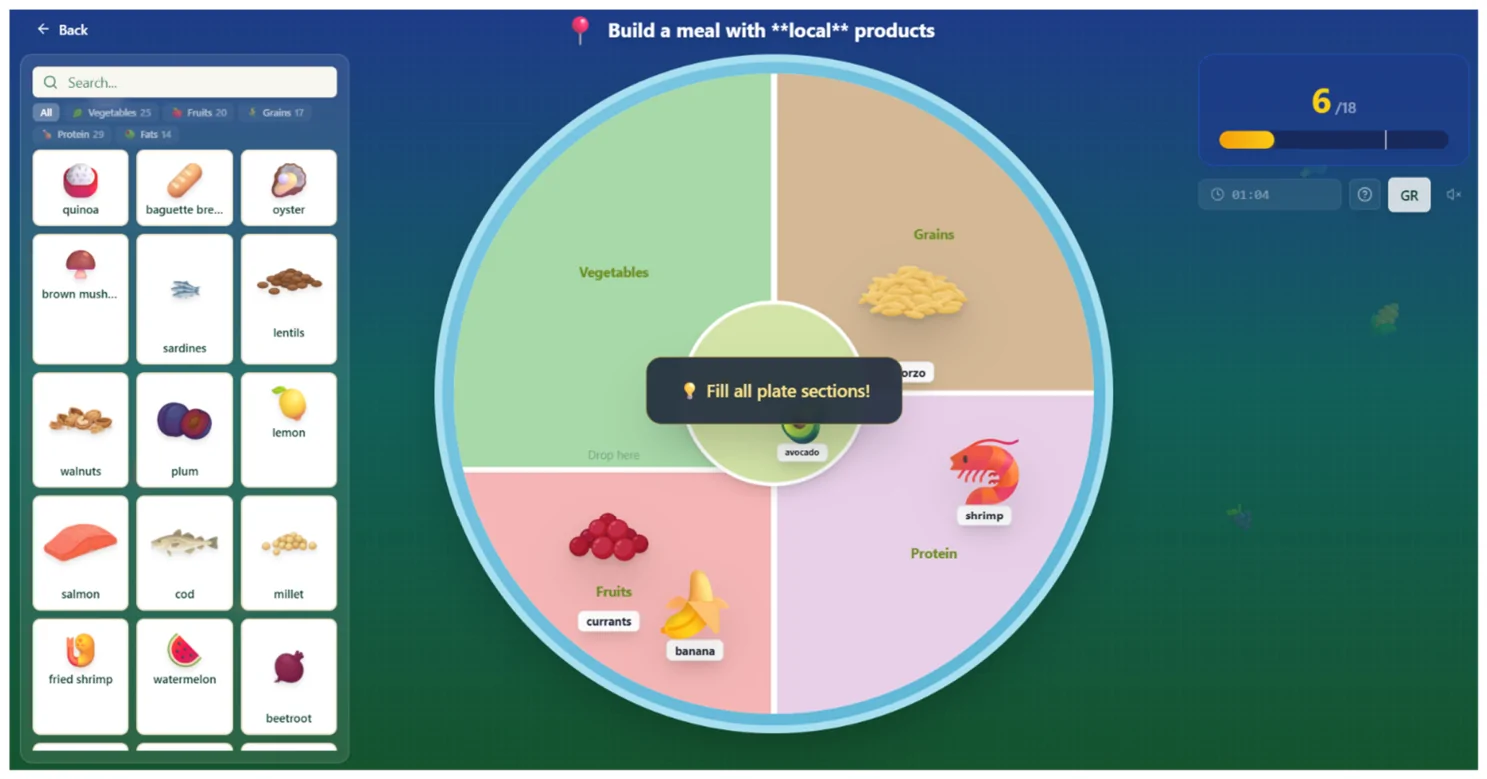
Main gameplay screen, including the food picker, the plate terrain, live score bar, challenge constraint, feature controls, and an inactivity overlay pop-up. The score displays the player’s live score relative to the pass threshold for the active challenge (e.g., 6/18) and is underlined for the maximum attainable challenge score.

Sustainable ePlate was developed as a single-player experience structured around nine sequential challenges, each representing a distinct indicator of SHDs. Across these challenges, the main interaction remains constant: the player must modify a plate that is pre-populated with suboptimal food items, replacing them with alternatives from a food picker (Figure 1). The objective is to score above a minimum threshold across three achievement tiers within each challenge.

Development of the game followed an iterative, user-centric approach over seven months (July 2025–January 2026), with co-creation sessions involving 12 students enrolled in secondary school grades 1–3 recruited from one of the participating schools. Students participated in three sequential formative sessions lasting approximately one hour. During the first session, students provided feedback on challenge structure, reward mechanisms, visual interface, and social features. Following each session, one researcher reviewed and prioritized the feedback, implementing iterative refinements to optimize usability and functionality across subsequent sessions. The process resulted in improvements to food images, tutorial instructions, score visualization, feedback messages, challenge structure, and reward indicators, alongside technical validation by 3 researchers.

#### 2.1.2. Food Database and Scoring

The game is based on a custom food database, developed to support both gameplay logic (scoring) and sustainability education. The database comprises 138 food items, spanning all five healthy eating plate (HEP) food groups (vegetables, fruits, grains, protein, fats). Foods are labeled by food group and sub-classified by protein or fat source (animal or plant).

All foods are further characterized by sustainability indicators (Table 1). Indicator selection was guided by three complementary considerations. First, the framework is aligned with the principal domains of sustainable healthy diets described in international guidance [10] and indicator reviews [6,7,8,9]. Within the game framework, nutritional balance and processing level represented the health/dietary domain; GHG emissions, water use, and land/soil impact represented environmental pressures; Mediterranean alignment, locality, and seasonality reflected food cultural context considerations; and the TCA-derived social indicators represented the social domain. Second, indicators had to be operationalized at the food level so that individual choices could directly affect gameplay under the HEP model. Third, indicators had to be interpretable and actionable for school-aged students and supported by evidence-based data through open-access datasets (the True Cost Accounting (TCA) database [38] based on the Sustainability Assessment of Foods And Diets (SAFAD) tool [39], and the Dutch National Institute for Public Health and the Environment (RIVM) LCA database [40] were identified for this purpose).

Two indicators address the dietary/health dimension: (i) dietary balance, based on the HEP model, and (ii) processing level, based on the NOVA classification system [41,42]. Three were selected to represent the environmental dimension: (i) carbon footprint, (ii) water use, and (iii) land/soil impact based on LCA data. Finally, four socio-cultural indicators—(i) social impact (TCA data), (ii) seasonality, (iii) locality, and (iv) cultural appropriateness—were country-specific, using appropriate food databases and food-based dietary guidelines. More specifically, in the current prototype, which was designed for Greek schools, seasonality is based on the latest Greek food-based dietary guidelines [43]. Locality was integrated at the national level rather than modeling regional differences. Foods commonly produced in Greece and typically available from Greek production were assigned the highest locality score. Foods generally sourced from other European or regional markets were assigned the intermediate score, while foods predominantly imported from more distant markets were assigned the lowest score. The score represented the likely origin of foods in the Greek market and did not use product-specific supply-chain traceability. Cultural appropriateness reflects alignment with the Mediterranean dietary principles [44] (Table S1 in Supplementary Material).

The nine challenges were not intended to constitute an exhaustive or consensus-weighted SHD index. Relevant considerations that were not modeled include affordability and economic access, biodiversity, animal welfare, food waste, packaging, food safety, supply-chain resilience, and product-specific provenance.

**Figure 2:**
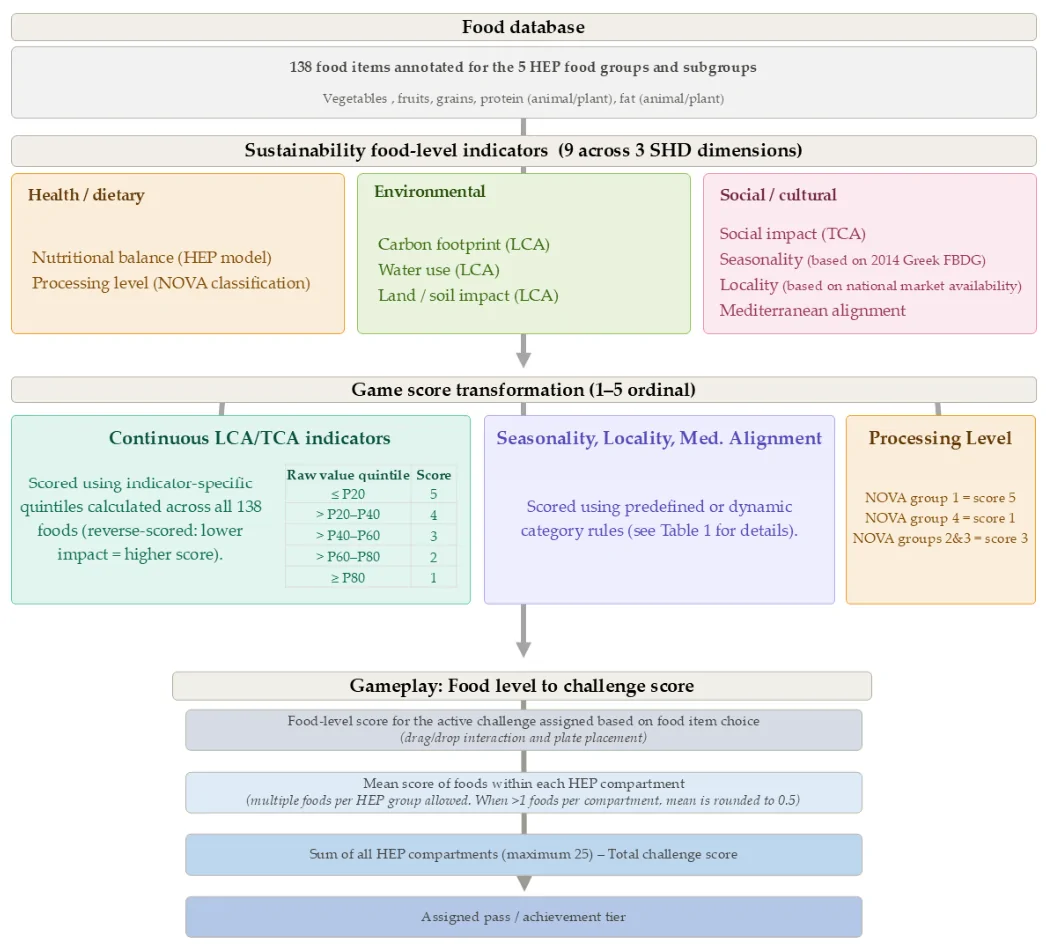
Schematic transformation of food-level sustainability indicators into Sustainable ePlate game scores.

To implement the game logic, all indicators are transformed into ordinal scores ranging from 1 to 5, where higher values consistently represent more desirable choices for the respective challenge. For each continuous environmental and social impact indicator, raw values were converted into ordinal scores using indicator-specific quintile thresholds calculated across all available foods within the dataset. Since lower raw impact values represented more desirable sustainability performance, scoring was reversed. Thresholds were calculated across the full food database, maintaining a common reference distribution across plate compartments and preserving more realistic relative differences in impact between foods belonging to different food groups. For categorical indicators, predefined scoring categories were used to preserve interpretability for school-aged users. Table 1 shows more information about the game’s logic and scoring, and Figure 2 illustrates the complete transformation to the scoring pipeline.

#### 2.1.3. Gameplay

**Figure 3:**
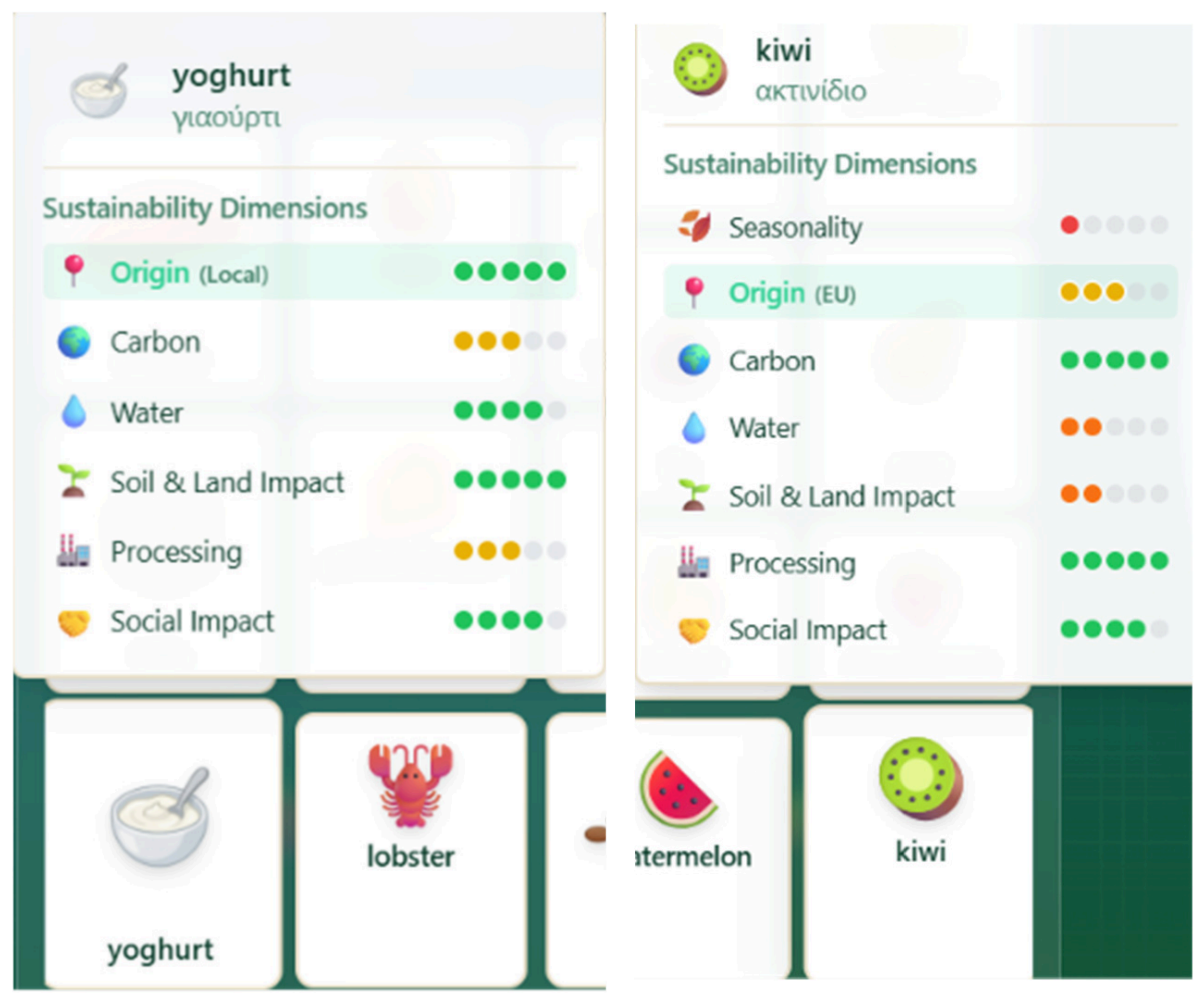
Food tooltips visible when hovering over food items (in picker or in plate) displaying the corresponding color-coded dot for each sustainability indicator.

Each plate that the user sees in a challenge is compartmentalized into five named sections corresponding to the five healthy eating plate (HEP) food groups [45]. The plate must be populated by at least one item per compartment to complete each challenge. Plates are pre-populated below the pass threshold, requiring players to improve meal composition through replacement. Hovering over any food (in picker or plate) opens a tooltip displaying the sustainability indicator scores on a five-level colored-dot scale (Figure 3).

The player advances the game by moving items (drag-and-drop) from the picker to the plate, either replacing or just adding them to the configuration. Incorrect placements (food items dropped to an incorrect food group) are rejected, and the food item is returned to the picker, followed by a corrective pop-up message identifying the correct group. After each successful placement or removal, the aggregate sustainability score is recomputed, and the progress bar is constantly updated.

For each challenge, the active indicator scores of the foods placed within each compartment are averaged and rounded to the nearest 0.5. The resulting scores for the five plate compartments are summed to produce a total plate score ranging from 5 to 25 points, with 25 representing the maximum score. The live score displays the player’s current score relative to the marked pass threshold (e.g., 6/18—Figure 1), while the full progress bar is scaled against the full 25-point score range.

Table 2 summarizes all interface components and their respective gameplay functions.

#### 2.1.4. Challenge Structure and Progression

**Figure 4:**
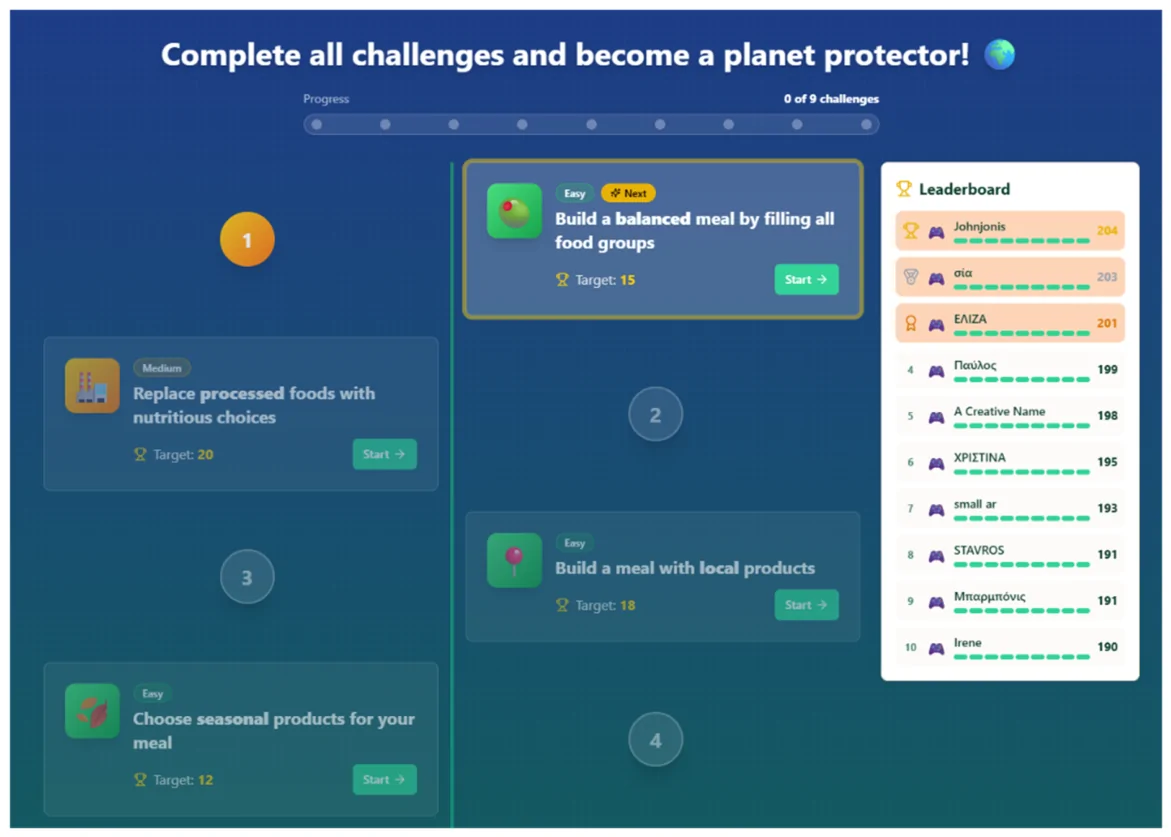
Journey tracker–main challenge screen and leaderboard (Greek names displayed on the leaderboard corresponded to the player names entered by participants).

Challenges are presented sequentially in the journey tracker (Figure 4). The first challenge asks players to achieve simple nutritional balance based on the HEP model. The remaining challenges target food-level SHD indicators: processing level, seasonality, locality, GHG emissions, water use, land/soil impact, social impact, and Mediterranean appropriateness. Challenges are unlocked sequentially, after preceding ones have been completed. Completed challenges are also available for replay attempts.

Progression is designed so that the first challenges focus on exposing individual dimensions in isolation (e.g., nutritional balance, processed foods), while later challenges require balancing trade-offs between competing dimensions. A challenge is complete when two conditions are simultaneously satisfied: (i) the plate is fully populated across all compartments (reflecting nutritional balance/adequacy) and (ii) the score equals or exceeds the pass threshold.

#### 2.1.5. Feedback

**Figure 5:**
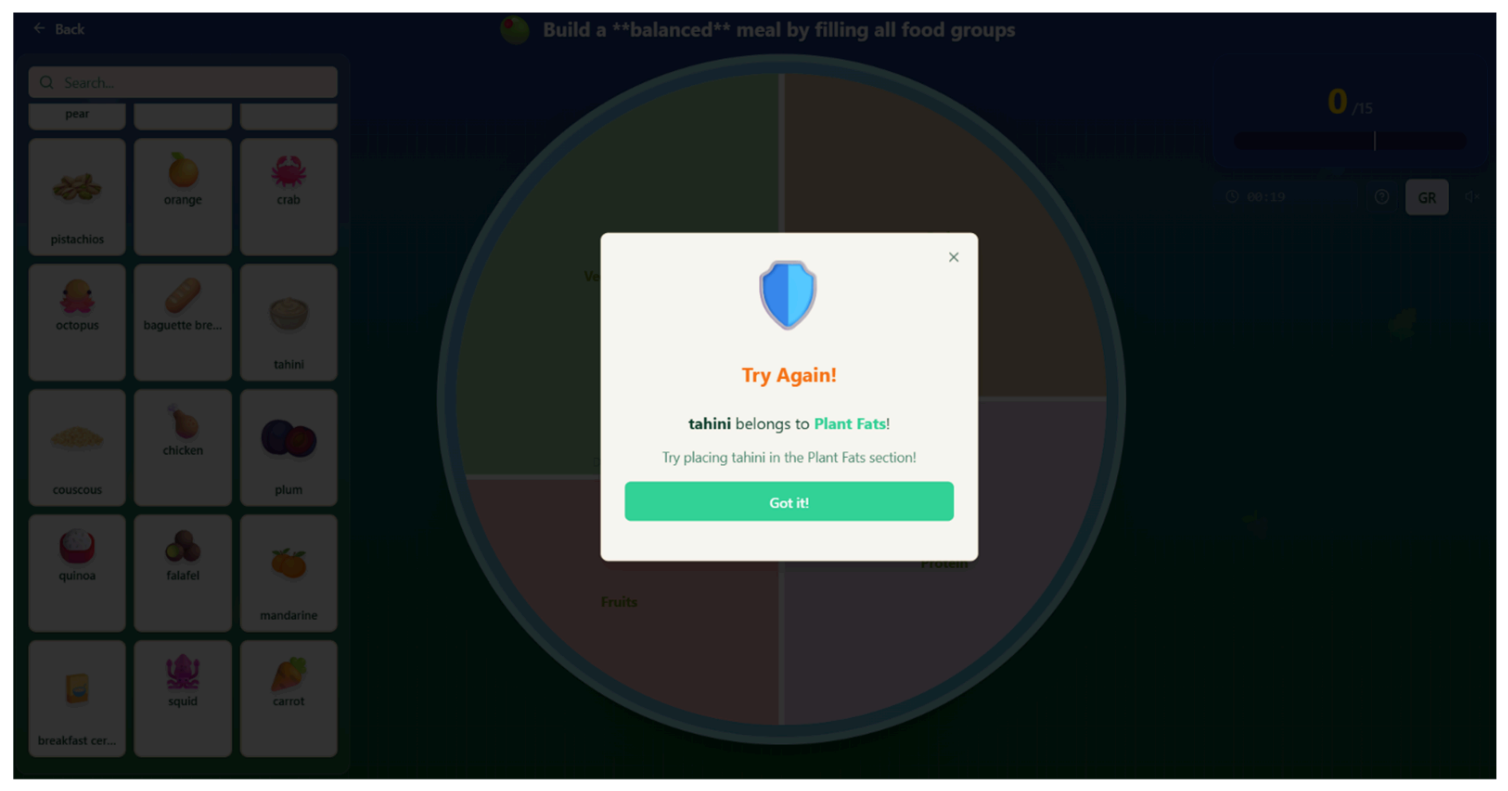
Feedback hint example (corrective food placement pop-up window).

Feedback is delivered continuously via the score-progress bar, correctively via food tooltips and rejection messages (Figure 5), and as a final summary via the challenge completion modal. Two scaffolding mechanisms support engagement: a 15 s inactivity hint overlay and a persistent help button available on every screen.

This approach serves as a dual educational function: providing immediate feedback on nutritional appropriateness while also enabling players to observe how food choices affected environmental, socioeconomic, cultural, processing-related, and dietary pattern dimensions of a meal.

#### 2.1.6. Deployment and Monitoring Dashboard

**Figure 6:**
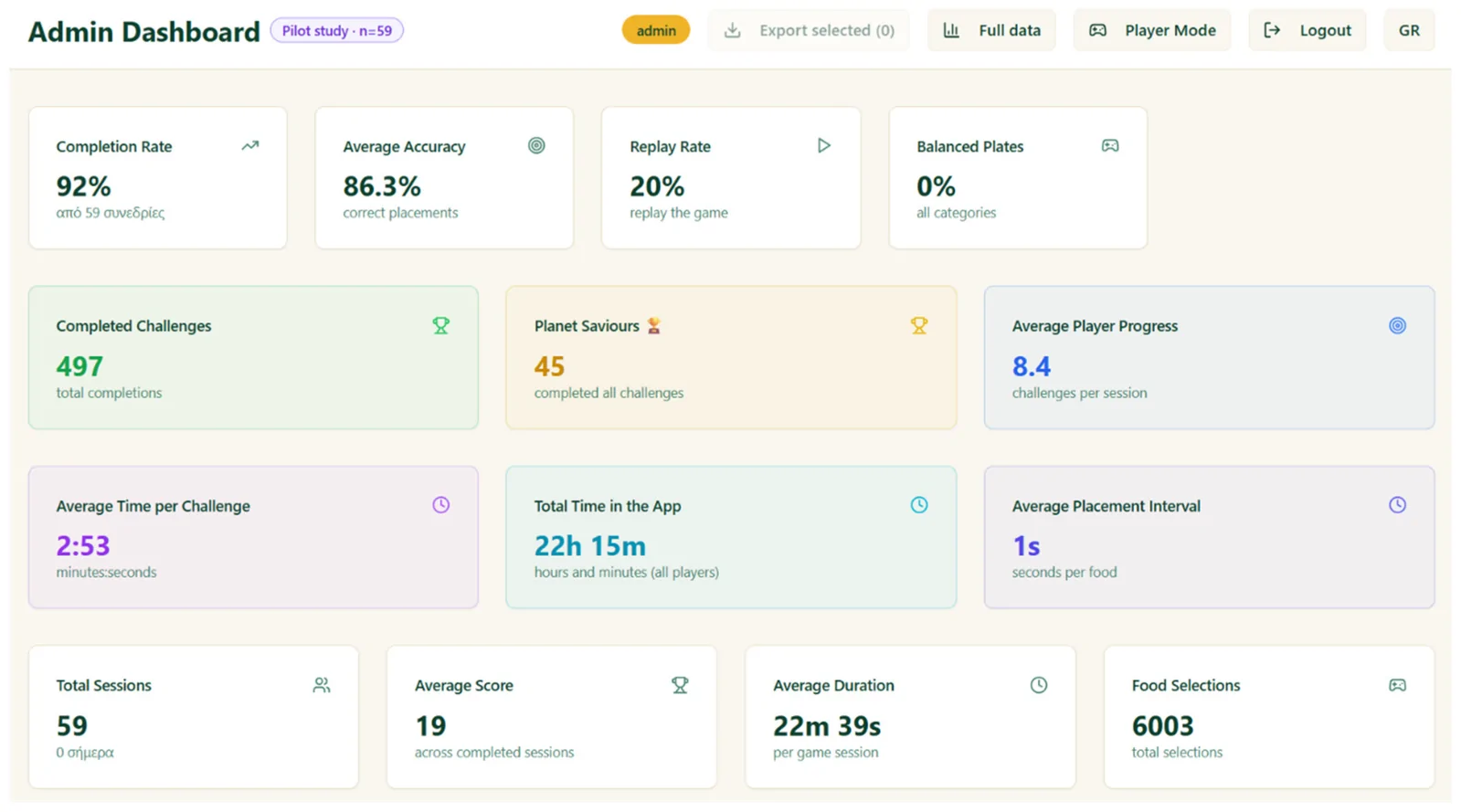
Monitoring dashboard.

The application was deployed on private servers via a Docker container architecture without external dependencies. The system’s backend database was designed to maintain transactional state for active gameplay sessions and capture interactions for learning analytics. Finally, a comprehensive tracking and monitoring dashboard (Figure 6) enabled detailed data logging, real-time analytics, player interactions, and dataset exports for further analysis.

### 2.2. Feasibility Testing Design

Following the game’s development and deployment, a feasibility study was conducted between March and May 2026 in schools in Northern Greece. The study took place exclusively during extracurricular school club activities.

In this feasibility study, participants completed a 60 min guided session that included using Sustainable ePlate and completing questionnaires. Sessions were conducted simultaneously in shared classrooms under the supervision of a researcher and a teacher after a brief thematic scope introduction. Gameplay records and questionnaire responses were collected individually; however, informal observation or communication could not be completely excluded. During gameplay, assistance was limited to technical support and clarification in case of unknown food items. Researchers and teachers were instructed not to recommend foods or provide answers relating to sustainability scores. Students used individual computer devices and were encouraged to progress through the challenge structure without discussion with peers. All participants encountered the same challenge order.

#### 2.2.1. Sampling and Participation Inclusion/Exclusion Criteria

Two schools in Northern Greece were recruited through convenience sampling. Students were eligible for inclusion if they were enrolled in primary (grades 4–6) or secondary (grades 7–9) education, enrolled in at least one school club, able to use a computer or tablet, and upon signed parental/guardian consent. Reasons for exclusion were absence from the intervention session, inability to use a computer, participation in the co-creation sessions, or consent withdrawal at any point. Since the study was designed to evaluate development and feasibility rather than educational effectiveness, the intended sample comprised a minimum size of *n* = 40, similar to other studies of this type [46] and considered appropriate for obtaining estimates for in-app metrics and initial user experience.

#### 2.2.2. Evaluation Measures

Feasibility was evaluated across three complementary aspects as shown in Table 3.

For all three aspects, in-app metrics were combined with the self-reported eGameFlow questionnaire [41], a 42-item validated instrument rated on a 7-point Likert scale across eight dimensions, including an overall enjoyment indicator on a 0–100 visual analog scale (VAS). The original English eGameFlow questionnaire was translated into Greek by three researchers. Discrepancies in translations were discussed by the team and resolved by consensus, prioritizing conceptual rather than strictly literal equivalence. Gameplay records (in-app metrics) and questionnaire responses were collected individually. However, informal observation or communication between classmates was not systematically recorded and could not be completely excluded.

#### 2.2.3. Statistical Analysis

All data were fully anonymized through pseudonymous player codes prior to analysis. Analyses were performed using R (version 4.4.1, R Core Team, 2024). Continuous variables (eGameFlow items and dimensions) are presented as means and standard deviations (SD), and categorical variables as frequencies or percentages. A one-sample *t*-test was used to examine whether eGameFlow scores differed from the neutral scale midpoint (test value = 4). Overall enjoyment was tested against the VAS midpoint (test value = 50). Cronbach’s alpha for the translated eGameFlow questionnaire was estimated as an indicator of internal consistency (since the Greek version was not subjected to a separate confirmatory factor validation). Effect sizes were reported as Cohen’s d with 95% confidence intervals. Normality of dimension and item-level scores was assessed using the Shapiro–Wilk tests. Where normality was not observed, Wilcoxon signed-rank tests were conducted. In-app game metrics (e.g., session duration, score) were summarized in descriptive statistics. Pearson correlations were tested between session duration, accuracy rate, placement interval time (in-app metrics), and some domain-level eGameFlow responses (overall enjoyment, concentration, immersion, and engagement) to explore convergence between behavioral and self-reported data.

## 3. Results

### 3.1. Participant Characteristics

The final analyzed sample comprised 59 completed gameplay sessions from two schools, of which 57 participants also completed the full eGameFlow questionnaire. Internal consistency was high for the full eGameFlow questionnaire (Cronbach’s α = 0.97) and all dimension subscales (Cronbach’s α > 0.87) (Table S2 in Supplementary Material). Table 4 presents the demographic characteristics of the study sample.

Participants were recruited from two schools, with 35 students from School 1 in Thessaloniki (59.32%) and 24 students from School 2 in Kilkis (40.68%). The mean age was 13.90 years (SD = 0.74), and the sample included 34 female students, corresponding to 57.63% of participants. All students who successfully participated in game sessions were enrolled in lower secondary school (grades 7–9).

### 3.2. Gamification Experience Results

eGameFlow dimension-level and overall enjoyment results are presented in Table 5. In terms of feasibility, acceptability was supported by high overall enjoyment and positive eGameFlow ratings across all dimensions.

Dimension-level mean scores ranged from 5.04 to 6.20, indicating that all dimensions were rated above the neutral midpoint. The highest-rated dimension was “Goal Clarity” (6.20 ± 1.07), followed by “Feedback” (6.09 ± 1.09), “Concentration” (6.06 ± 0.91), and “Knowledge Improvement” (6.06 ± 1.08), and demonstrated the highest effects, ranging from d = 1.90 to d = 2.28. These findings suggest that participants perceived the game as clear in its objectives, supportive in terms of feedback, and relevant to the learning task. The knowledge improvement score reflects perceived knowledge support rather than an objective measure of learning. Lower mean scores and effects were observed for “Immersion and Engagement” (d = 0.66) and “Social Interaction” (d = 0.86), suggesting that although these aspects were evaluated positively, they were less strongly endorsed than the more task- and learning-oriented dimensions. Overall enjoyment was high (82.54 ± 16.33), consistent with the positive dimension-level results.

Detailed descriptive statistics for all eGameFlow items are presented in Table S3 in the Supplementary Material. Most item-level scores were above the scale midpoint, although affective and visceral immersion items showed weaker and more variable endorsement.

### 3.3. Feasibility Results

Overall, the available self-reported and in-app metrics provided evidence across two of the complementary feasibility aspects. Usability and engagement were supported by high ratings for goal clarity, feedback, concentration, and autonomy/control, together with an overall placement accuracy of 86.27% across 6003 food selections and measurable decision and interaction times. Implementation feasibility was supported by the completion of the full nine-challenge sequence in all 59 recorded gameplay sessions, a mean session duration of 22.39 ± 12.68 min, and successful capture of interaction data across sessions. Table 6 summarizes the key in-app behavioral metrics extracted from the monitoring dashboard for *n* = 59 unique gameplay sessions.

Across all sessions (full 9-challenge sequence), mean duration was 22.39 ± 12.68 min, suggesting participants were able to complete the game within a manageable time frame for a school-based activity [47]. Overall placement accuracy was 86.27%, with both correct and incorrect placement events recorded across sessions. Participants attempted 546 challenges (including repeats to increase score) and earned a total of 384 badges. The average placement interval (17.57 ± 7.28s) and hesitation times (1.06 ± 0.30s) indicate that the interaction design allowed sufficient time for decision-making without preventing participants from completing the game.

Challenge completion scores varied across challenge objectives. The highest average scores were achieved in the “Processed food” challenge (22.22 ± 1.90), followed closely by “Land and soil impact” (22.13 ± 1.91), “Social impact” (21.95 ± 1.36), and “Locality” (21.82 ± 2.69). Moderate scores were recorded for the “GHG” (20.59 ± 2.28) and “Water saving” (20.56 ± 1.85) challenges. The lowest average performances were recorded for the “Mediterranean” (19.06 ± 1.59) and “Seasonality” (14.21 ± 2.76) challenges, with the latter recording the greatest variability and the lowest mean score across all challenges.

Exploratory correlations between selected in-app metrics and domain-level eGameFlow self-reported responses did not reveal any significant associations. Output is available under Table S4 in the Supplementary Materials.

## 4. Discussion

This study presents the design and feasibility evaluation of Sustainable ePlate, a web-based serious game developed to support multidimensional SHD school-based interventions. Overall, the findings provide initial support for its feasibility across acceptability, usability, engagement, and implementation. High enjoyment and positive eGameFlow ratings indicate good acceptability, with the most favorable self-reported dimensions relating to goal clarity, feedback, concentration, and knowledge improvement, while affective immersion and social interaction were less strongly endorsed. Placement accuracy and recorded interaction metrics further support usability and engagement with the game. Implementation feasibility was demonstrated by completion of the full game sequence in all recorded sessions and capture of intended in-app interaction metrics during gameplay.

All sessions completed the full nine-challenge sequence within a mean of 22.39 ± 12.68, indicating that the game could be appropriate within scheduled sessions for school-based delivery. Wide variability in session completion time may reflect inter-individual differences in food literacy, familiarity with digital tools, or exploratory replay consistent with self-paced digital learning environments as seen in other studies [48].

Acceptability and game experience, assessed via overall enjoyment through eGameFlow’s VAS, was 82.54 (SD = 16.33), which compares favorably with reported findings in serious game evaluations in educational settings [49,50]. This is consistent with broader evidence from systematic reviews demonstrating that game-based learning environments tend to produce high acceptability ratings among school-aged populations, particularly when interventions are delivered in a structured, guided format [25,30]. Dimension-level eGameFlow scores ranged from 5.04 to 6.20 on a seven-point scale, with all dimensions exceeding the midpoint. The highest-rated dimensions were “Goal Clarity” (6.20), “Feedback” (6.09), “Concentration” (6.06), and “Knowledge Improvement” (6.06), suggesting that game features were well-developed. High “Goal Clarity” and “Feedback” ratings reflect the sequential challenge structure, tutorial, and live score-progress bar, consistent with the role of immediate feedback as a core behavior change technique associated with improved learning outcomes and sustained engagement [25,26]. High “Knowledge Improvement” scores suggest that students perceived the game to support understanding and application of the content. Since no objective knowledge measure was administered at baseline, knowledge acquisition cannot be established, although existing evidence supports that serious games can effectively support active knowledge acquisition in nutrition and health education contexts [31]. The “Immersion and Engagement” dimension yielded the lowest aggregate score and within-dimension heterogeneity across all eGameFlow dimensions (5.04 ± 1.59). While items capturing absorption were moderately endorsed (5.49), affective and visceral involvement items received the lowest scores and highest variability across the entire questionnaire (4.47 and 4.53, respectively). This may suggest that while the game was sufficient to occupy participants’ attention and direct their cognitive effort, it did not produce deeper affective states associated with flow. This is not uncommon in evaluations of serious games with explicitly educational objectives, where functional engagement with learning tasks takes precedence over hedonic or affective involvement [51]. This suggests that participants may have attended to and interacted with the task without experiencing deeper affective or narrative absorption commonly associated with entertainment-focused games [52]. Several features of the present evaluation may have contributed to this pattern, including the guided classroom context, the short single-session exposure, the absence of a developed narrative, and the only task-related feature, a drag-and-drop interaction. “Social Interaction” was rated moderately (5.30), with the highest endorsement for the item “Cooperation in the game is helpful to learning” (5.77), despite the game being a single-player experience, which may reflect classroom-level peer presence and informal cooperation rather than use of in-app social features. The relatively favorable rating of this item may reflect informal peer interaction in the shared classroom setting. This highlights an opportunity for future development, including peer comparison or collaborative features that have been shown to enhance engagement and motivation in school-based digital interventions, particularly among adolescents [53,54]. Overall, findings suggest that the game was perceived as clear, responsive, educationally useful, and capable of sustaining students’ attention. However, large effect size values should be interpreted with caution due to the single-group post-intervention design, as they indicate strongly favorable user-experience ratings rather than evidence of intervention effects.

Compared to other nutrition-focused serious games which similarly reported positive user experiences and incorporated objective pre–post nutritional-knowledge measures [35,36], Sustainable ePlate differs in both scope and evaluation stage: it addresses a broader set of sustainable-diet indicators, including nutritional, environmental, social, and food system-related dimensions, whereas the current study was restricted to development, acceptability, and implementation feasibility. Therefore, the favorable eGameFlow ratings observed here can be compared with those studies as user-experience findings but should not be interpreted as evidence of comparable educational effectiveness.

In-app interaction metrics provided complementary evidence of usability and meaningful engagement. A placement accuracy rate of 86.27% across 6003 food selections indicates that the food group classification system and plate interface were sufficiently clear for students to use effectively. Meanwhile, the presence of incorrect placements indicates that the game retained an appropriate level of challenge [55]. This pattern is compatible with an interface in which progression required interaction with the educational content, rather than functioning as superficial gamification elements [56,57]; however, the present study did not directly test the pedagogical contribution of individual game mechanics. The alignment between intervention activities and outcomes is particularly important in public health nutrition interventions, where engagement must be balanced with accurate communication of complex dietary and sustainability concepts [58]. The mean placement interval and hesitation times demonstrate that food placement was not instantaneous, but its determinant cognitive meaning cannot be validated from the present data alone. Results may suggest participants engaged in some processing rather than impulsive responding, consistent with reflective decision-making around food classification, a supportive precursor to connecting food choices with broader health and sustainability considerations [59], and would mirror findings from digital nutrition platforms where decision latency may proxy engagement depth [24]. However, it cannot be determined from these data alone, and future studies should examine association with baseline literacy, placement accuracy, and objective learning outcomes before interpretation as a marker of engagement depth. In-app metrics and self-reported engagement measures did not correlate in this sample. Given the current sample size, this should not be interpreted as evidence that behavioral and self-reported engagement are unrelated, as the study had limited power to detect small effects. Beyond supporting the technical feasibility of the current implementation, the monitoring architecture enables future evaluation of within-session processes that are usually unavailable from post-intervention questionnaires, such as time and accuracy across repeated challenges, breadth of food exploration, challenge abandonment, and within-player changes in placement interval and hesitation times. These measures could be examined for validation as potential indicators of engagement, task mastery, strategy development, or learning progression. Future studies should evaluate whether these in-app measures can predict objectively measured pre–post change, delayed retention, food-choice preference, or sustained participation, while accounting for baseline food literacy and digital familiarity.

Challenge completion times suggest preliminary insights into different cognitive demands; “Mediterranean” (3.50 min) and “Water saving” (3.09 min) were the most demanding challenges, while “Social Impact” (1.31 min) and “Locality” (2.32 min) were completed quickly. Differences between completion times should not be interpreted as differences in intrinsic challenge difficulty, since baseline knowledge was not measured and therefore performance may also reflect differences in students’ prior familiarity with these topics. Score distributions may additionally have been influenced by the initial plate composition, or the number and range of available scoring alternatives. Future designs should consider assessing baseline familiarity with the intervention context.

A core contribution of Sustainable ePlate is its attempt to integrate the multidimensional nature of SHDs within a single interactive platform, addressing a structural gap identified in the literature (22). By integrating multiple distinct sustainability indicators covering environmental, health, socioeconomic, and cultural dimensions, the platform provides students with exposure to the full breadth of SHD dimensions in a progressive, scaffolded format. The integration of data from the TCA database, national food-based dietary guidelines, an established classification system for processing level, and dynamic metadata effectively translates complex food system information into age-appropriate, interactive content. The behavioral monitoring infrastructure further positions the game as a dual-purpose research tool capable of supporting learning analytics research alongside its primary educational function, consistent with calls for richer engagement data capture in digital nutrition intervention research [60,61].

Some limitations related to the feasibility evaluation and system design must be acknowledged. First, the feasibility study involved a convenience sample of 59 students from two schools in Northern Greece, limiting the generalizability of findings. All analyzed students attended lower-secondary education and had to be enrolled in at least one school club, which may have resulted in students with greater motivation, school engagement, or digital familiarity than the broader student population. The findings therefore cannot be directly generalized to primary school students, other school types, or independently delivered classroom use. Second, the study used a single-arm feasibility design without a comparison group, objective baseline or post-intervention knowledge assessment, or longitudinal follow-up. Therefore, it cannot determine whether the game can lead to changes in knowledge, attitudes, dietary behavior, or retention. The eGameFlow knowledge improvement dimension reflects perceived rather than objectively verified learning. In addition, all sessions were conducted in a shared classroom under guided researcher and teacher supervision, which may have influenced enjoyment, hesitation, help-seeking, persistence, and questionnaire responses (although recorded individually). Participants’ awareness of taking part in a supervised activity potentially created Hawthorne effects [62] and socially desirable questionnaire responses [63]. In addition, because students participated simultaneously in a shared classroom, incidental peer observation or informal communication could not be completely excluded. Furthermore, zero recorded access to the help function and leaderboard during sessions may reflect the constrained guided format rather than genuine disinterest or need in the features. Finally, the study was limited to evaluating the feasibility and user experience of the intervention and did not assess its educational effectiveness. In particular, the eGameFlow knowledge improvement dimension represents participants’ perceived learning experience and should not be interpreted as evidence of objectively measured knowledge gain. In addition, in-app metrics such as hesitation time and other interaction variables have not yet been validated as indicators of cognitive engagement or learning. Placement interval may reflect processes such as deliberation, uncertainty, food-search time, interface exploration, or differences in digital literacy. Their interpretation as learning-analytics measures requires validation against objective educational outcomes. Without objective measures of sustainable healthy diet knowledge, attitudes, food-choice behaviors, or knowledge retention, no conclusions can be drawn regarding its educational impact, which remains to be explored. Future work should examine immediate and retained learning using objective measures of sustainable food literacy, alongside attitudes, behavioral metrics, and food-choice outcomes, in a design including validated baseline, immediate, and follow-up post-intervention measures that would allow educational effects to be distinguished from positive user-experience ratings.

It should also be noted that the 1–5 ordinal scoring system and Greek/Mediterranean cultural calibration of this implementation of Sustainable ePlate limit direct transferability to other age groups and regions without adaptation of food items, scoring parameters, and localization. In addition, regional variation within Greece, product-specific origin, affordability, biodiversity, food waste, and other potentially relevant dimensions were not fully represented. Adaptation to other countries or populations would require revision of the food list to reflect local consumption patterns, dietary guidelines, locality and seasonality, cultural appropriateness, use of country-specific LCA/TCA data, and language, followed by local expert review and user testing. While expert validation was conducted internally within the multidisciplinary development team, independent external validation, potentially via structured expert-consensus methods (e.g., Delphi), would strengthen the validity of the modeling framework.

Future research should prioritize controlled educational effectiveness through larger sample trials, examining pre–post changes in knowledge, attitudes, and practices through validated measures following exposure to Sustainable ePlate immediately and at longitudinal follow-up. Studies should also explore whether in-app metrics are associated with learning outcomes, to determine whether gameplay analytics can be meaningful markers of engagement or learning.

## 5. Conclusions

Sustainable ePlate demonstrates the feasibility of integrating multidimensional sustainability modeling, theory-informed gamification, and real-time behavioral monitoring within a web-based serious game for school-based interventions. Participants reported high overall enjoyment and favorable ratings of goal clarity, feedback, concentration, and perceived knowledge improvement, while the platform successfully captured detailed in-app interaction data. These findings support acceptability, usability, and implementation feasibility in the evaluated setting; however, they do not establish objective learning, dietary behavior change, or knowledge retention. The system provides a foundation for integrating multiple sustainable healthy diet dimensions within a school-based intervention activity. Future research should examine the relationships between engagement, learning processes, and educational intervention outcomes through larger controlled and longitudinal studies, via validated pre- to post-game session measures, and explore associations between in-app interaction metrics to provide insights into how interaction patterns relate to learning and behavior change.

## Figures and Tables

**Table 1:** Food-level indicators, data sources, original units, and transformation into game scores.

Indicator	Source	Original Unit	Game Logic/Scoring
Carbon footprint	LCA	kg CO_2_-eq/kg	Reverse-scored using indicator-specific quintiles across the food database, with lower emissions receiving higher scores (1–5)
Water use	LCA	m^3^/kg	Reverse-scored using indicator-specific quintiles across the food database, with lower emissions receiving higher scores (1–5)
Land/soil impact	LCA	kg 1,4-DCB-eq/kg	Reverse-scored using indicator-specific quintiles across the food database, with lower emissions receiving higher scores (1–5)
Social impact	TCA	€/kg	Reverse-scored using indicator-specific quintiles across the food database, with lower emissions receiving higher scores (1–5)
Nutritional meal balance	Healthy eating plate model	Food-group classification for vegetables, fruits, grains, protein foods, and fats	Implemented through correct placement into the HEP compartments (not directly scored or accounting for HEP food group ratios)
Processing level	NOVA	NOVA groups 1–4	Collapsed into a three-tier reversed score. NOVA group 1 received the highest score (5); NOVA group 4 received the lowest score (1), and NOVA groups 2 and 3 were combined into the intermediate score = 3
Seasonality	FDB/FBDG	Monthly availability	Assigned a dynamic 1–5 score according to seasonal availability in Greece during the month of gameplay
Locality	FDB/FBDG	Local/EU/Imported	Three-tier score according to likely origin in the national Greek market. Imported = 1, regional or EU = 3, local to Greece = 5.
Cultural Appropriateness	Mediterranean alignment	Dynamic *	Mapped to a 1–5 score based on alignment with Mediterranean dietary principles

* detailed computation in Table S1. Supplementary Material.

**Table 2:** Central gameplay screen interface components.

Element	Location	Function
Food picker	Left side of main gameplay screen	Presents available food items grouped by category
Compartmentalized plate	Center of main gameplay screen	Placeholder visual plate that receives food items based on food groups
Score-progress bar	Right side of main gameplay screen	Displays the live meal score (0–25) with markers for three level thresholds
Food tooltips	Overlay, hovering over food items (main gameplay screen)	Display a colored 5-dot indicator for each sustainability indicator
Journey tracker	Challenge navigation screen	Lists the nine-challenge sequence, their current state (locked/unlocked), and tier achieved per challenge
Inactivity overlay	Centered pop-up over gameplay plate	Appears after 15s of inactivity with a contextual hint
Completion modal	Centered pop-up upon challenge completion	Reports the achieved tier, awards the corresponding badge, and offers replay or return-to-tracker actions
Player dashboard	Journey tracker (challenge navigation screen)	Displays the player’s badge collection and allows for controlled score resets
Leaderboard	Journey tracker screen	Shows player rankings by named avatars

**Table 3:** Mapping of feasibility aspects to self-reported eGameFlow and in-app indicators.

Feasibility Aspect	Definition	Source	
		eGameFlow	In-App Metrics
Acceptability	Overall enjoyment and game engagement	Overall enjoyment eGameFlow dimension scores	-
Usability and engagement	Students’ ability to use the game mechanics, engagement level, apply rules, and interact meaningfully with the content.	Concentration Goal clarity Challenge Autonomy/control Feedback Immersion/engagement	Session duration Food selections Correct/incorrect placements Accuracy rate Placement interval time Hesitation time Drag-drop duration Challenge completion time Challenge scores
Implementation	Delivery of the game in the school setting, completion within a single school session, technical functionality, and successful data capture.	-	Total recorded sessions Session completion rate Challenge completion time Successful capture of interaction metrics

**Table 4:** Demographic and school characteristics of participants who completed the eGameFlow questionnaire and the overall game-session sample.

Characteristic	Completed eGameFlow (*n* = 57)	All Game Sessions (*n* = 59)
Age, years (mean ± SD)	13.88 ± 0.76	13.90 ± 0.74
Female, *n* (%)	34, 60%	34, 57.63%
Lower secondary (grades 7–9), *n* (%)	57, 100%	59, 100%
Upper primary (grades 4–6), *n* (%)	-	-
School 1, *n* (%)	33, 57.90%	35, 59.32%
School 2, *n* (%)	24, 42.10%	24, 40.68%

SD: standard deviation.

**Table 5:** eGameFlow dimension-level results: descriptive statistics, one-sample *t*-test, and effect sizes (*n* = 57).

Dimension	Mean	SD	95% CI (Mean)	t	95% CI (d)	Cohen’s d
Concentration	6.06	0.91	5.82–6.30	17.21	1.78–2.77	2.28
Goal Clarity	6.20	1.07	5.91–6.48	15.51	1.59–2.51	2.05
Feedback	6.09	1.09	5.81–6.38	14.57	1.49–2.37	1.93
Challenge	5.61	1.31	5.26–5.96	9.30	0.88–1.57	1.23
Autonomy/Control	5.79	1.33	5.44–6.14	10.13	0.98–1.70	1.34
Immersion and Engagement	5.04	1.59	4.62–5.46	4.95	0.37–0.94	0.66
Social Interaction	5.30	1.51	4.89–5.70	6.48	0.55–1.16	0.86
Knowledge Improvement	6.06	1.08	5.77–6.35	14.37	1.46–2.34	1.90
Overall Enjoyment, VAS	82.54	16.33	78.21–86.88	15.04	1.54–2.44	1.99

SD: standard deviation, CI: confidence interval. VAS: Visual Analogue Scale. Shading indicates scoring on a 0–100 VAS, whereas the remaining eGameFlow dimensions were scored on a 1–7 scale.

**Table 6:** In-app interaction metrics summary (*n* = 59) in counts, means, and standard deviations.

Metric	*n =* 59
**Summary metrics**	
Completed sessions (*n*)	59
Session duration (minutes)	22.39 ± 12.68
Correct food placements (*n*)	5179
Incorrect food placements (*n*)	824
Average accuracy rate (%)	86.27
Average placement interval (seconds) ^a^	17.57 ± 7.28
Average hesitation/drag-drop (seconds) ^b^	1.06 ± 0.30
**Challenge completion score (mean ± SD)**	
Nutrition balance	N/A
Mediterranean	19.06 ± 1.59
Processed foods	22.22 ± 1.90
Water saving	20.56 ± 1.85
GHG	20.59 ± 2.28
Seasonality	14.21 ± 2.76
Locality	21.82 ± 2.69
Land and soil impact	22.13 ± 1.91
Social impact	21.95 ± 1.36
**Challenge completion time in minutes (mean ± SD)**	
Nutrition balance	N/A
Mediterranean	3.50 ± 2.72
Processed foods	2.91 ± 1.47
Water saving	3.09 ± 2.40
GHG challenge	2.67 ± 1.73
Seasonality	2.40 ± 1.53
Locality	2.32 ± 1.05
Land and soil impact	2.05 ± 1.22
Social impact	1.31 ± 0.74
**Interactions**	
Total challenges attempted (*n*)	546
Total food selections (*n*)	6003
Total badges earned (*n*)	384
Total help access (*n*)	0
Total leaderboard access (*n*)	0

SD: standard deviation; **^a^** Average placement interval: time elapsed between two consecutive food placements (selections–*n*); **^b^** Average hesitation/drag-drop: time interval from selecting the food to placing it on the plate; N/A: not captured for the nutritional balance challenge.

## Data Availability

The datasets generated and analyzed during this study are available from the corresponding author upon reasonable request, subject to ethical restrictions on participant data.

## Supplementary Materials

The following supporting information can be downloaded at https://www.mdpi.com/article/10.3390/nu18172812/s1. Table S1: Rules applied per food group; Table S2: Cronbach’s alpha for the 42 eGameFlow questionnaire items, and per dimension; Table S3: eGameFlow subscale item-level descriptive statistics (*n* = 57); Table S4: Correlations (Pearson r [95% CI]) between in-app metrics and eGameFlow domains (*n* = 57).

## Abbreviations

The following abbreviations are used in this manuscript:

GHG	Greenhouse Gases
HEP	Healthy Eating Plate
LCA	Life Cycle Assessment
SHD	Sustainable Healthy Diets
TCA	True Cost Accounting
VAS	Visual Analog Scale
