# Supplementary material for: Development and Feasibility Study of Sustainable ePlate: A Web-Based Serious Game for Multidimensional Sustainable Healthy Diet Interventions in Schools
Source: Nutrients. 2026 Aug 27;18(17):2812. doi: 10.3390/nu18172812 (PMC13567481; doi:10.3390/nu18172812)
Supplement: Supplementary file 1 [file nutrients-18-02812-s001.zip › nutrients-4473612-supplementary.pdf]

Supplementary Material

Mediterranean score

*The score aims to server purely **game transformation purposes**, following the same game mechanics previously used by the players across earlier rounds. Therefore, to maintain food-level annotation (scoring), rules were applied separately to each of the five different food groups.*

Table S1. Rules applied per food group

Group	Values	MedDiet rationale*	Per-value Med score (1–5)
**Protein**	• Legume<br>• Fish<br>• Poultry, eggs, dairy<br>• Red_meat<br>• Processed meat	• Plant & fish proteins are core<br>• Red/processed meats are limited groups	legume = 5<br>fish = 4<br>poultry_egg_dairy = 3<br>red_meat = 2<br>processed_meat = 1
**Fats**	• Olive oil & olives<br>• Nuts & seeds<br>• Plant oils<br>• Animal fat	• Olive oil & nuts are the MedDiet fat backbone<br>• Plant based oil are preferred over animal fats	olive = 5<br>nuts_seeds = 5<br>other_plant (plant oils) = 3<br>animal = 1
**Grains**	• Whole<br>• Mixed<br>• Refined	• Whole grains are the pyramid base<br>• Refined are limited	whole = 5<br>mixed = 3<br>refined = 1
**Vegetables**	-	Seasonal & local produce is encouraged	dynamic = mean of seasonality & origin scores
**Fruits**	-	Seasonal & local produce is encouraged	dynamic = mean of seasonality & origin scores

** The scoring rules were reviewed internally by three dietitians and one informatics member of the development team for consistency, nutritional plausibility, and compatibility with the game logic. No independent external consensus procedure was conducted.*

Internal consistency of the translated eGameFlow questionnaire

Table S2. Cronbach's alpha for the 42 eGameFlow questionnaire items, and per dimension

Scale / Dimension	# items	Cronbach's α
**Full eGameFlow questionnaire**	42	0.968
**Concentration**	6	0.876
**Goal Clarity**	4	0.918
**Feedback**	5	0.937
**Challenge**	6	0.882
**Autonomy / Control**	3	0.923
**Immersion & Engagement**	7	0.925
**Social Interaction**	6	0.920
**Knowledge Improvement**	5	0.883

Item level results of the translated eGameFlow questionnaire

Table S3. eGameFlow subscale item-level descriptive statistics (*n*=57)

Dimension	Mean	SD
**Concentration**		
Most of the gaming activities are related to the learning task	6.26	1.11
No distraction from the task is highlighted	5.98	1.27
Generally speaking, I can remain concentrated in the game	6.18	1.26
I am not distracted from tasks that the player should concentrate on	6.05	1.03
I am not burdened with tasks that seem unrelated	5.86	1.08
Workload in the game is adequate	6.05	1.16
**Goal Clarity**		
Overall game goals were presented in the beginning of the game	6.28	1.22
Overall game goals were presented clearly	6.28	1.10
Intermediate goals were presented in the beginning of each scene	6.02	1.30
Intermediate goals were presented clearly	6.21	1.15
**Feedback**		
I receive feedback on my progress in the game	6.07	1.19
I receive immediate feedback on my actions	6.04	1.19
I am notified of new tasks immediately	6.07	1.32
I am notified of new events immediately	6.11	1.13
I receive information on my success (or failure) of intermediate goals immediately	6.19	1.23
**Challenge**		

The game provides "hints" in text that help me overcome the challenges	6.04	1.32
The game provides "online support" that helps me overcome the challenges	5.70	1.55
The game provides video or audio auxiliaries that help overcome challenges	4.70	2.10
The difficulty of challenges increased as my skills improved	5.72	1.53
The game provides new challenges with an appropriate pacing	6.16	1.21
The game provides different levels of challenges tailored to different players	5.35	2.01
**Autonomy/Control**		
I feel that I can use strategies freely	5.72	1.49
I feel a sense of control and impact over the game	5.75	1.35
I know next step in the game	5.89	1.44
**Immersion & Engagement**		
I forget about time passing while playing the game	5.49	1.72
I become unaware of my surroundings while playing the game	4.91	2.02
I temporarily forget worries about everyday life while playing the game	5.09	1.75
I experience an altered sense of time	5.49	1.65
I can become involved in the game	5.30	1.74
I feel emotionally involved in the game	4.47	2.23
I feel viscerally involved in the game	4.53	2.18
**Social interaction**		
I feel cooperative towards other classmates	5.25	1.84
I strongly collaborate with other classmates	5.11	1.78
Cooperation in the game is helpful to the learning	5.77	1.42
The game supports social interaction between players	5.19	1.93
The game supports communities within the game	5.16	1.92
The game supports communities outside the game	5.30	1.76
**Knowledge Improvement**		
The game increases my knowledge	6.14	1.30
I catch the basic ideas of the knowledge taught	6.21	1.16
I try to apply the knowledge in the game	6.23	1.10
The game motivates the player to integrate the knowledge taught	6.11	1.32
I want to know more about the knowledge taught	5.61	1.62

SD: standard deviation

Exploratory correlations between in-app user data and self-reported experience measures

Table S4. Correlations (Pearson r [95% CI]) between in-app metrics and eGameFlow domains (*n*=57)

In-app metrics	eGameFlow self-reported domains		
	Overall Enjoyment	Concentration	Immersion & Engagement
Session Duration	−0.13 [−0.38, 0.14]	−0.02 [−0.28, 0.24]	−0.02 [−0.28, 0.24]
Accuracy Rate	−0.05 [−0.31, 0.21]	0.11 [−0.16, 0.36]	0.04 [−0.22, 0.30]
Placement Interval	0.16 [−0.11, 0.40]	0.15 [−0.11, 0.40]	0.03 [−0.23, 0.29]

All $p > .23$ ($\alpha = .05$)
